# Attitudes and Beliefs of Primary Care Providers in New Mexico About Lung Cancer Screening Using Low-Dose Computed Tomography

**DOI:** 10.5888/pcd12.150112

**Published:** 2015-07-09

**Authors:** Richard M. Hoffman, Andrew L. Sussman, Christina M. Getrich, Robert L. Rhyne, Richard E. Crowell, Kathryn L. Taylor, Ellen J. Reifler, Pamela H. Wescott, Ambroshia M. Murrietta, Ali I. Saeed, Shiraz I. Mishra

**Affiliations:** Author Affiliations: Richard M. Hoffman, University of New Mexico School of Medicine, University of New Mexico Cancer Center, Albuquerque Veterans Affairs Medical Center, Albuquerque, New Mexico; Andrew L. Sussman, Robert L. Rhyne, University of New Mexico Cancer Center and Department of Family Medicine and Community Medicine, Albuquerque, New Mexico; Christina M. Getrich, Department of Anthropology, University of Maryland, College Park, Maryland; Richard E. Crowell and Ali I. Saeed, University of New Mexico School of Medicine and University of New Mexico Cancer Center, Albuquerque, New Mexico; Kathryn L. Taylor, Georgetown Lombardi Comprehensive Cancer Center and Georgetown University Medical Center, Washington, DC; Ellen J. Reifler and Pamela H. Wescott, Healthwise, Boise, Idaho; Ambroshia M. Murrietta, Clinical and Translational Science Center, University of New Mexico Health Sciences Center, Albuquerque, New Mexico.

## Abstract

**Introduction:**

On the basis of results from the National Lung Screening Trial (NLST), national guidelines now recommend using low-dose computed tomography (LDCT) to screen high-risk smokers for lung cancer. Our study objective was to characterize the knowledge, attitudes, and beliefs of primary care providers about implementing LDCT screening.

**Methods:**

We conducted semistructured interviews with primary care providers practicing in New Mexico clinics for underserved minority populations. The interviews, conducted from February through September 2014, focused on providers’ tobacco cessation efforts, lung cancer screening practices, perceptions of NLST and screening guidelines, and attitudes about informed decision making for cancer screening. Investigators iteratively reviewed transcripts to create a coding structure.

**Results:**

We reached thematic saturation after interviewing 10 providers practicing in 6 urban and 4 rural settings; 8 practiced at federally qualified health centers. All 10 providers promoted smoking cessation, some screened with chest x-rays, and none screened with LDCT. Not all were aware of NLST results or current guideline recommendations. Providers viewed study results skeptically, particularly that 95% of abnormal LDCT results were false positives, the need to screen 320 patients to prevent 1 lung cancer death, and the small proportion of minority participants. Providers were uncertain whether New Mexico had the necessary infrastructure to support high-quality screening, and worried about access barriers and financial burdens for rural, underinsured populations. Providers noted the complexity of discussing benefits and harms of screening and surveillance with their patient population.

**Conclusion:**

Providers have several concerns about the feasibility and appropriateness of implementing LDCT screening. Effective lung cancer screening programs will need to educate providers and patients to support informed decision making and to ensure that high-quality screening can be efficiently delivered in community practice.

## Introduction

The National Lung Screening Trial (NLST) showed that lung cancer screening with low-dose computed tomography (LDCT) significantly reduced lung cancer deaths among heavy smokers compared with screening with chest x-ray (1). The US Preventive Services Task Force (USPSTF) subsequently issued a B recommendation supporting LDCT screening (2). The recommendation is important because the Affordable Care Act mandates first-dollar coverage for preventive services graded A or B by the USPSTF (3). In February 2015, the Centers for Medicare and Medicaid Services (CMS) proposed that evidence is sufficient to provide annual LDCT screening for patients and in centers meeting eligibility criteria (4). The American Lung Association (5) and American Cancer Society (6) also support LDCT screening. However, the American Academy of Family Physicians determined that the evidence was insufficient to recommend for or against lung cancer screening with LDCT (7).

Translating results of an efficacy trial conducted largely in academic medical centers into routine community practice may be challenging. Nearly all participants in the NLST were white, their socioeconomic status was higher than the general population, and they were adherent to recommended testing (1). US population data show marked racial/ethnic and socioeconomic disparities in lung cancer mortality, prevalence of smoking, stage at diagnosis, and adherence to cancer screening (8,9). New Mexico, the setting for our study, is a large, sparsely populated minority–majority state (non-Hispanic whites make up less than 50% of the population) characterized by low socioeconomic status and limited health care resources (10). 

Documenting the perspectives of providers caring for racially/ethnically and socioeconomically diverse populations is necessary for planning screening implementation. However, few studies have evaluated physician attitudes and practices regarding LDCT lung cancer screening (11–14), and US studies were conducted before NLST results and screening recommendations were published. Therefore, we interviewed primary care clinicians practicing in New Mexico to characterize their knowledge, attitudes, and beliefs about LDCT lung cancer screening.

## Methods

### Study setting and sample

We conducted qualitative in-depth, semistructured interviews with primary care provider members of the Research Involving Outpatient Settings Network (RIOS Net), the practice-based research network of New Mexico (15). Approximately one-third of RIOS Net members practice in federally qualified community health care centers that predominantly serve Hispanic patients. By using a purposeful sampling approach, we identified rural and urban clinicians whose patients met criteria for LDCT screening. The University of New Mexico Health Sciences Center’s Human Research Protections Office approved the study. We obtained consent by reviewing the consent form with each provider prior to the interview. 

### Data collection

We developed an interview guide (Appendix) that focused on the domains of knowledge, attitudes, and beliefs regarding LDCT lung cancer screening that we believed to be relevant to the potential uptake and implementation of new primary care services, perceived patient receptivity to screening, perceived decisional influences, barriers and facilitators, perceived patient receptivity to smoking cessation and relapse prevention, perceived decisional influences, and views about cultural factors related to screening and smoking behaviors among Hispanic and other racial/ethnic minority and underserved populations. Additionally, we asked about the feasibility of effectively engaging patients in screening and smoking cessation discussions. 

We conducted the interviews from February through September 2014. We mailed the materials about the study and lung cancer screening to primary care providers up to 1 week before the interview. Materials included the informed consent form, a summary of the NLST study design and results, a summary of lung cancer screening guidelines developed by national organizations (American Cancer Society, USPSTF, and the American Lung Association), and a pictogram that graphically displayed outcomes (true–false positives rates, absolute mortality benefits) of lung cancer screening based on the NLST. Providers could refer to these materials during the interview. The digitally audio-recorded interview lasted about 60 minutes and was transcribed. All but 1 interview was conducted in person; providers received a $50 merchandise card for participating.

### Data analysis

We used a content-driven immersion and crystallization approach to data analysis, which consisted of a systematic iterative process of transcript content review to identify themes of importance (16). Each research team member independently read sets of 2 or 3 transcripts to identify preliminary findings. This process enabled us to modify the interview guide to confirm emerging themes, disconfirm anomalous results, and pursue unanticipated issues. Through the use of these verification strategies, the lead qualitative analysts (A.M., C.G., A.S.) created an initial coding structure that was continually revised during the data collection until the team reached consensus (17). We then imported the transcripts into NVivo 10 (QSR International), a qualitative data analysis software program, for coding. We grouped data into broad categories to guide theme development. After coding all transcripts, we queried the database by coding categories for a more refined level of interpretive analysis. The team iteratively reviewed these reports to identify and crystallize major analytic themes. We reached thematic saturation after interviewing 10 primary care providers.

## Results

The 10 primary care providers included 2 physician assistants; all were family medicine clinicians. Eight practiced at federally qualified health centers and 4 in rural settings; 4 were women.

**Current practices for smoking cessation and lung cancer screening.** Providers reported usually inquiring about tobacco use and estimated that about 10% to 40% of their patients would meet LDCT screening eligibility criteria, with highest rates among men (Table, quote 1). However, providers noted that male smokers were least likely to come in for preventive health care, because they usually accessed the health care system only when they were sick. Providers reported counseling smokers about cessation, identifying resources such as quit lines, and prescribing nicotine replacement therapy, bupropion, or varenicline. Some ordered a baseline chest x-ray for smokers, particularly for those with lung disease, expecting that abnormal results could serve as a scare tactic. One provider believed that ordering a chest x-ray alone might prompt some smokers to consider quitting.

**Table:** Primary Care Provider Comments About Lung Cancer Screening, New Mexico, February to September, 2014

Domain	Provider Comments
**Current practices for smoking cessation and lung cancer screening**	1. “Yes. I think a lot of people do [fall into screening eligibility category]. Um . . . in that age group I would say it’s probably a good . . . upwards of 25%.”
	2. “Yeah, I actually had no idea. I read this guideline stuff right before . . . like earlier this morning and I was like, ‘I had no idea the USPSTF even like recommended this.’ I knew nothing about it. In fact, when you sent the email about the study, I was like, ‘we’re supposed to be screening for lung cancer?’ I had no idea at all.”
**Interpreting evidence from the National Lung Screening Trial **	3. “I mean, you are putting someone at harm I guess, I mean, that’s a lot of radiation exposure. There’s not a huge difference between the group that was screened and not screened. Three people is not a huge difference, and the fact that 365 people had a false positive. It’s like way more people had a false positive than like a real positive and for a screening test it doesn’t seem like a great test.”
	4. “I think I feel very ambivalent about somebody doing one of these studies. I think the biggest thing for me is that we’ve got . . . it’s like we have 3 fewer deaths. Right? Out of per thousand. But then per those thousand people, 3 people are having major complications.”
	5. “And then just physical access to getting the test. I mean, here’s a patient of mine who I say, ‘hey, you’re gonna need to go here, wherever, to get this,’ [and the patient says] ‘okay, let’s see, buy my medicine for two weeks, or pay for gas to go do this. Let’s see, okay should I get that ultrasound, well I guess we can go on tortillas and beans for the week, for me and the two kids, or I can do that to try and make it work to get the money to do this particular exam.’”
**Perspectives on screening guidelines**	6. “I see what the grade recommendation is. Is it a ‘C’? It’s not that great. Then I won’t do it for everybody, like if it was an ‘A’ then I should be doing that. That’s how that integrates into my practice.”
	7. “It comes up a lot with a lot of our screening tests that are recommended. Like they’re level B evidence or something and you could do it but it’s just like it’s Pandora’s Box so you do it and it’s not a great screening test, um, where like the number needed to screen is like also in the hundreds and then it . . . there’s all these false positives and procedures.”
	8. “It’s something I worry about . . . is this person feeling like, ‘Oh well, I’ve got a clean bill of health. My lungs are fine. I might as well puff away.”
	9. “Well, I think the harm of testing, you know, the false-positive rate and what that actually means is the number-one concern with any test we do. I think we’ve just gone through this or are still going through this with PSA [prostate specific antigen] and prostate cancer screening, and we’re still trying to figure out how to have that conversation.”
	10. “I feel like the more I learn about screening, the more I’m reticent and kind of, you know, everything coming out around mammography and obviously we no longer recommend PSAs and prostate cancer screening. I feel like I needed some time to absorb it and find the time to read everything and try to figure out what’s going on, but it’s definitely been on my radar.”
**Implementing screening**	11. “[W]ithout having further information about this, and with the limited reading I’ve done on this, I probably um . . . it’s not . . . because it’s not a guideline that’s in stone yet, I probably would not be inclined to offer it to every single person who is in this category.”
	12. “Definitely my initial thought looking through the data was like, ‘I’m not gonna do this . . . yeah, seems like way too much . . . the radiation exposure is a lot per year and the cost of this kind of screening is humongous. It’s not like we’re doing x-rays on everybody, we’re doing CTs.”
	13. “We shouldn’t be screening people if we know that they can’t afford it or if it’s going to represent a massive financial burden and then follow-up. I hadn’t even thought about the fact that maybe they won’t be covering the work-up afterwards, which is probably true because the work-up is going to be pretty expensive. That really worries me.”
	14. “It’s a matter of where you put your resources. We could all come up with our priority list, much of which would be probably above this [screening]. Basic medications, other tests that patients are having, physical therapy patients are having to pay 40% of the co-pay. It would just seem from a policy perspective that those kind of more low-tech approaches may be a better way to use resources, in the big picture.”
	15. “We really need to come up with probably a totally different model for prevention and screening activities that is parallel to primary care in terms of acute and chronic care, which is not to say that the primary care clinician cannot or should not be doing the best they can with their limited time with the patient, but in addition to that there should be some very different model for prevention and screening activity. As you know in many countries around the world that there’s just a whole separate system for prevention and screening, which again, can be paired with primary care, acute and chronic care, but to, to keep thinking that we can just keep adding to a list of things that, you know, the primary care provider’s supposed to do in their 20-minute visit is, is just silly and naïve and spinning our wheels and actually creating various problems.”
	16. “System changes have left providers in a worse position to address important things like prevention screening, education, medication management, self-management . . . all these things which are pretty critical to really improving the health of patients.”
**Screening counseling**	17. “A lot of it depends on how we’re able to boil it down for them . . . help them understand and make their decision. We influence that whether we want to or not. We influence their decision based on how we present it. It could be a slippery slope. I find that folks are like, ‘I don’t want to know and then be worrying about something or freaked out.’ Having this kind of discussion takes a high level of health literacy. It's hard to explain these kind of numbers.”
	18. “I think that is the most basic level of understanding, ‘hey, there’s a test and doctors can do this test and it’s like, yes or no, you have something or you don’t.’ And then the next level of understanding is what does that something mean? That means I could have a nodule and from there on I would say the majority of my patients would have no concept. It’s like they tune out or it’s just too difficult for them to wrap their head around that.”
	19. “I think if there were a way to play a video in the room because we are gonna have computers in the room. If we get to that level of technical ability then that’d be nice. Some of my patients can’t read so that would be another benefit.”
**Ethical considerations**	20. “People are actually desperate to receive quality care. It’s actually depressing that most of our patients have heard about good and helpful and important health care that apparently some people receive out in the world but that they don’t get to receive because of access problems. So folks are desperate and anxious for the day to come when they’ll have access to the kinds of things they see on TV.”
	21. “Yeah, and even if it’s not necessarily like a cost to the patient, it would be, you know, someone’s paying for it . . . other people who haven’t smoked are paying the premiums for people who like have smoked and are now getting these CTs every year.”

No clinician ordered LDCT scans for lung cancer screening, and none reported any patient demand for such screening. Some were unaware of LDCT screening, whereas others reported LDCT was just “getting on their radar” (Table, quote 2). Among providers who order chest x-rays, some explained that they believed they were following national guidelines and others were uncertain whether screening with LDCT was superior to using chest x-ray.

**Interpreting NLST evidence.** Not all providers were aware of the LDCT results. When we presented summaries of the trial a few were positive about screening, but most viewed the data with some skepticism. They perceived the absolute mortality risk benefit to be small; the number of patients who would need to be screened in order to prevent 1 lung cancer death was seen as high. Other providers wanted to see evidence that screening was efficacious for different populations and indicated hesitance about initiating screening based solely on 1 trial. There was considerable concern about the potential harm of screening. Providers often cited the high rate of false-positive results and their downstream consequences. Subsequent diagnostic testing, particularly invasive procedures, further affect patients by complicating risks from diagnostic procedures, missing work, arranging transportation to an imaging center, and facing insurance copayments. Some wanted to see a lower false-positive rate before widely adopting LDCT. Concerns about the health risks from long-term radiation exposure would deter some from ordering LDCT screening (Table, quotes 3–5).

**Perspectives on screening guidelines.** Not all providers were aware of the recent changes to national cancer screening guidelines to include LDCT; a few believed that guidelines recommended screening with a chest x-ray. Although some were inclined to follow screening recommendations (“even if one life is saved, it is a good procedure”), others felt that the guidelines were still too nebulous. The USPSTF B rating was problematic for some providers, who tended to aggressively counsel patients only for A-rated preventive services. They expected a convincing standard of evidence to support guidelines leading to rapid changes in practice, which they felt was not met for lung cancer screening (Table, quotes 6–7). Providers welcomed the clear selection criteria related to age and smoking history but were concerned about continuing screening until age 80, an upper age limit that went beyond the NLST inclusion criteria. They also believed that the guidelines did not sufficiently address the appropriateness and safety of screening patients with multiple chronic conditions. Additionally, they noted that USPSTF recommendations did not factor in potential outcomes following a negative screening result, including a false sense of reassurance and continued use of tobacco products (Table, quote 8).

Providers discussed their experiences with the evolving changes to mammography and prostate specific antigen screening guidelines, which led to increased scrutiny about the value of all screening tests. Some believed that lung cancer screening needs to be viewed in the broader context of the overall risks, benefits, and costs for all screening programs. They also pointed out that survival benefits might be more attributable to improved treatments than to screening efforts (Table, quotes 9–10).

**Implementing screening.** Most providers were cautious and ambivalent about offering lung cancer screening, preferring to take a “wait and see” approach (Table, quotes 11–12). However, given the USPSTF recommendation, many expected that screening would eventually be implemented at their practice sites. Providers felt that they will be obligated to offer screening if it becomes a performance measure. Furthermore, some were concerned about the potential legal implications associated with not having offered either chest x-ray or LDCT to a patient who subsequently develops lung cancer.

Providers identified many potential barriers to implementing screening. One concern related to following up on abnormal test results given the range of possible diagnostic and surveillance testing options. Radiologists would need to provide primary care providers clear guidance about addressing positive findings, but providers were also worried about whether New Mexico had the infrastructure to support the high-quality screening program required by guidelines. Screening rural patients annually would be particularly problematic given the lack of local technology and resulting travel burdens. Most were concerned about the costs for follow-up testing after positive screening results and the costs of treating cancer, which could be prohibitive for many patients (Table, quote 13). Additionally, they recognized that patients, particularly in rural areas, would incur substantial costs related to travel and lost income from taking time off from work. Although many potentially eligible clinic patients were using Medicaid or Medicare, others were self-paying, either uninsured or uninsurable (undocumented immigrants). Providers indicated that their screening decisions would be heavily influenced by socioeconomic status.

Integrating an additional screening program was perceived as potentially overloading an already stressed primary care system (Table, quote 14). Providers described facing many competing patient demands and already lacking time to engage in counseling about valuable preventive care services. One physician noted that effectively addressing LDCT screening in primary care would require a system redesign (Table, quote 15). However, providers also recognized that clinics, especially federally qualified health centers, were already overwhelmed with the new requirements of the Affordable Care Act and meaningful use of electronic health records (Table, quote 16). Many providers indicated that incorporating any new preventive service would be a slow process.

**Screening counseling.** Providers wrestled with counseling about screening because they considered screening to be a complex issue, particularly regarding abnormal results that did not clearly suggest cancer. Presenting the idea that an abnormal test result is not definitive (“we don’t know what it means”) and that further testing is required could be challenging given the low literacy of many of the patients and the limited available discussion time during clinic visits. One respondent noted that patients do not understand risk, particularly the concept of absolute versus relative risk, so would have difficulty making informed decisions about lung cancer screening. Providers did mention the importance of providing concrete information so that patients could relate to estimates for risk and benefit. However, providers were also worried about the challenges of helping patients understand the potential down-stream consequences of screening without scaring them. Providers were themselves uncertain about the follow-up of abnormal test results and were uncomfortable advising patients about lung cancer screening. Some indicated that the challenges in describing the risks and benefits might actually prevent them from discussing screening. Finally, providers recognized that current screening guidelines could commit eligible patients to decades of screening. However, they would support patients who wanted to opt out and understood, based on their experiences with breast and prostate cancer screening, that guidelines could change (Table, quotes 17–19).

**Ethical considerations.** Lastly, providers raised several potential ethical issues. One provider sensed that offering screening in underserved communities represented a form of health equity because rural patients are often excluded from latest advances (Table, quote 20). However, others considered it ethically wrong to offer screening to patients who cannot afford follow-up care. Another provider, who was unwilling to offer screening, was concerned about the societal costs of “free” screening, because nonsmokers are paying premiums for smokers to receive screening (Table, quote 21). A few providers questioned the value of devoting limited health care resources to LDCT screening among older, hard-core smokers. They instead advocated for focusing cancer control efforts on the young to achieve greater societal benefit by creating environmental barriers and financial disincentives to starting or continuing smoking.

## Discussion

This qualitative study of primary care providers practicing in New Mexico raised important concerns about implementing lung cancer screening. Providers felt that the state might have limited capacity to provide the high-volume, high-quality lung cancer screening and treatment centers required to support screening programs. Access and cost would likely be important patient barriers to implementing screening, and respondents also were concerned about the feasibility of integrating yet another screening recommendation into routine practice. Several providers were not fully aware of the NLST results or of the major lung cancer screening guidelines, and they indicated that they would need guidance in advising patients about the screening process. They also identified ethical issues related to resource allocation.

Providers were skeptical about incorporating another preventive service into their practice, not only because of time constraints but also because of the complexity of explaining and coordinating screening and follow-up. Although USPSTF recommendations carried weight, respondents indicated that a performance measure would make screening a higher priority. CMS support for LDCT will likely have a major impact on whether health care systems and organizations, such as the National Center for Quality Assurance, focus on screening by developing a measure for the Health Care Effectiveness Data and Information Set (HEDIS).

A consistent message in the guidelines is that providers should help patients make informed screening decisions. However, not all our respondents were aware of the evidence on lung cancer screening or of national guideline recommendations. These findings are similar to a national survey conducted in 2006–2007 showing practice patterns at variance with evidence and guidelines. For example, some providers ordered sputum cytology or screened never smokers, although at that time of the study, guidelines did not recommend lung cancer screening (11,12). Educating primary care providers about evidence and recommendations will be a necessary step in implementing lung cancer screening. Although some providers suggested continuing medical education training, an approach that has shown some effectiveness for changing practice (18), a more practical approach might be to develop provider decision support tools that summarize the clinical evidence and guideline recommendations for lung cancer screening and provide guidance on offering smoking cessation counseling and referrals. The USPSTF developed a 1-page information sheet on lung cancer screening for providers (19). Given the complexity of screening issues, the American Cancer Society called for primary care providers to develop competency in shared decision making, which they describe as a rapidly emerging obligation for primary care (6). Implementing shared decision making may also allay provider concerns (20) about malpractice and unwarranted patient requests (12,13). The American Lung Association is developing a toolkit to assist at-risk patients in discussions with physicians (though not specifically with primary care providers) (5), and a patient decision aid is being evaluated in a Patient-Centered Outcomes Research Institute grant (21). However, multidisciplinary teams comprising radiologists, pulmonologists, and oncologists with expertise in evaluating, diagnosing, and treating abnormal lung findings will likely need to assume responsibility for supporting decision making once an abnormality is detected through LDCT screening, including decision making about invasive diagnostic procedures and surveillance testing.

Our respondents brought up important concerns about the costs of screening for individual patients. Although the costs of initial screening tests are covered under the Affordable Care Act, health care systems and health departments will still need to address the uncertain coverage of surveillance imaging, invasive diagnostic procedures, and cancer treatment that could burden the patient. Respondents were also concerned about the overall costs of screening programs and questioned whether New Mexico has the necessary resources to implement lung cancer screening. The societal costs of a lung cancer screening program could be prohibitive given the estimated 8.7 million US adults eligible for screening (22). An economic analysis suggested that the national costs of implementing LDCT screening would be $1.3 to $2.0 billion dollars annually; while screening (with 75% uptake) could avoid up to 8,100 premature lung deaths cancers, the additional cost of screening to avoid 1 death would be about $240,000 (23). Based on NLST findings, the estimated cost-effectiveness of LDCT screening would be $81,000 per quality-adjusted life year (24). However, the estimate was sensitive to model assumptions; costs could be higher depending on how screening is implemented in community settings.

The majority of our respondents believed that interventions to control lung cancer should be linked to interventions to control tobacco use (14). A cost–utility analysis indicated offering smoking cessation interventions with annual screening would increase the cost-effectiveness of lung cancer screening by 20% to 45% (25). Interestingly, some respondents raised ethical concerns about allocating resources to screening for lung cancer rather than to controlling tobacco use, their reasoning being that helping smokers, particularly younger smokers, to quit would be a more ethical method of controlling the incidence of lung cancer than screening smokers to learn whether they already have lung cancer.

Our study has some limitations. The sample size was small, although we quickly reached saturation in our themes. We interviewed providers caring for underserved minority patients, and the attitudes and beliefs of providers in our study might differ from those of providers practicing in different settings. However, given that most US primary care providers are not practicing in settings comparable with the academic centers that participated in the NLST, our findings are likely to be broadly generalizable. In addition, we distributed materials about LDCT screening to providers before the interviews, which may have influenced their responses. However, none of our respondents were screening with LDCT, and we found it necessary to provide this material to produce meaningful discussions. Because we provided information only about lung cancer screening, respondents could not readily compare study findings and guidelines with other screening programs. Nonetheless, we presented material adapted from guidelines issued by prominent organizations, such as the USPSTF, American Cancer Society, and American Lung Association, which clinicians routinely review to guide screening practices. Furthermore, clinicians indicated awareness of the controversies surrounding prostate and breast cancer screening programs.

Our findings have some important implications for lung cancer screening in New Mexico. The American Cancer Society guideline explicitly advised offering screening only if eligible patients could access an appropriate screening and treatment center (6). If New Mexico has the necessary infrastructure to implement screening, it will likely be in a limited number of urban settings, which would create access problems for rural patients. Screening may also be fragmented, with opportunistic screening occurring in settings without ready access to diagnostic procedures and treatment. As encouraged by the USPSTF (2) and required by CMS (4), New Mexico should consider creating a registry to track screening practices and outcomes, including abnormal LDCT findings, invasive diagnostic testing and complications, and treatment outcomes. Resource use and cost data should also be collected to determine the most cost-effective strategies. Such a registry would help determine how well NLST results translate into practice and identify quality improvement efforts to address gaps.

Given limited resources, screening programs will need further efficiencies, including identifying high-risk patients who would most benefit from screening. A post-hoc analysis of NLST data suggests that targeting screening to the 3 highest risk quintiles (based on age, body mass index, family history of lung cancer, smoking history, and emphysema diagnosis) would account for 88% of screening-prevented lung cancer deaths (26). Conversely, providers will need to avoid screening patients who do not meet criteria or who are too sick to benefit. Previous surveys suggest that some primary care providers would not routinely consider a patient’s age, tobacco use, or exposure to tobacco smoke when deciding whether to recommend screening (11,12,14).

We conducted what we believe is the first US qualitative provider study following publication of NLST results and issuance of the USPSTF lung cancer screening guidelines. Implementing lung cancer screening will be challenging, particularly in underserved areas. The program will require ensuring access to the necessary infrastructure, closely adhering to screening guidelines, addressing costs for both patients and health care systems, and supporting shared decision making. Even if screening programs are not established, providers will need help counseling patients who request screening or who have undergone opportunistic testing. Policy makers will need to consider establishing registries to track screening practices and outcomes and will also need to determine whether allocating resources to screening is the most cost-effective approach to controlling the burden of lung cancer.

## Appendix Primary Care Provider Interview Guide

This file is available for download as a Microsoft Word document
