# Erratum, Vol. 12, July 9 Release

**DOI:** 10.5888/pcd16.150112e

**Published:** 2019-11-07

An author error in the wording of the Results section of the Abstract was brought to our attention in the article entitled “Attitudes and Beliefs of Primary Care Providers in New Mexico About Lung Cancer Screening Using Low-Dose Computed Tomography,” which was published in *Preventing Chronic Disease* on July 9, 2015. Authors have revised the original wording to clarify the meaning.

The original wording was as follows:

Providers viewed study results skeptically, particularly the 95% false-positive rate, the need to screen 320 patients to prevent 1 lung cancer death, and the small proportion of minority participants

Authors revised the wording as follows:

Providers viewed study results skeptically, particularly that 95% of abnormal LDCT results were false positives, the need to screen 320 patients to prevent 1 lung cancer death, and the small proportion of minority participants.

The article was corrected on October 23 and appears online at https://www.cdc.gov/pcd/issues/2015/15_0112.htm. We regret any confusion or inconvenience this error may have caused.

