# Supplementary material for: Attitudes and Beliefs of Primary Care Providers in New Mexico About Lung Cancer Screening Using Low-Dose Computed Tomography
Source: Prev Chronic Dis. 2015 Jul 9;12:E108. doi: 10.5888/pcd12.150112 (PMC4509091; doi:10.5888/pcd12.150112)
Supplement: Supplementary file 1 [file 15_0112_Appendix.docx]

**Appendix: Primary Care Provider Interview Guide**

**I. Lung Cancer Screening**

1. I’d like to start by first hearing about rates of tobacco use among your older—55 to 79 year old—patients?
   1. Approximately what % would you estimate are current smokers?
   2. Are there differences between males and females?
2. We’re going to talk more about this group of current and long-term former smokers in the practice but now I’d like to talk about lung cancer screening with low dose CT. What have you heard about these recent developments? Do you have any thoughts or opinions about the pros/cons of lung cancer CT screening with your patients?
   1. Do you currently screen middle-aged men with a smoking history for abdominal aortic aneurysms?

***[Interviewer to review FAQ sheets--NLST Summary and Guideline Fact Sheets (ACS, ALA, USPSTF)*** I have some information about some of the research that has led to current screening recommendations as well as initial guidelines that I would like to briefly review with you.**]**

1. What are your thoughts about this information and screening recommendations?

Prompts: I would like to focus on a few specific areas with you

- - 1. What are your views about the relative risks and benefits to LDCT screening for your patients?

*(Possible risks: fear, radiation exposure, false positives/incidental findings, complications from diagnostic follow up)*

*(Possible benefits: enhance tobacco cessation efforts, reduce lung cancer deaths, peace of mind)*

- - 1. Thinking from the perspective of your patients, how receptive would they be to LDCT screening? How feasible is this for your patients?
    2. Considering again the rates of smoking that we talked about earlier, about what % of these patients would be eligible for LDCT?

**II. Tobacco Cessation Counseling Efforts**

1. We’re going to talk some more about how best to offer LDCT screening to your patients but before we do that, I’d like to take a step back and hear about your current tobacco cessation counseling efforts. Can you tell me what leads you to spend time with a patient to counsel them to stop smoking?
   - 1. What strategy(ies) have you found to be most effective? (*probe whether medications have been effective)*
     2. What tools, resources, information do you have available to support smoking cessation efforts?
     3. Do you involve other providers/staff in these efforts? Referral to community based programs?
2. In your experience, what leads patients to stop smoking? *(Possible influences: family/friend; proximity to someone close diagnosed with lung disease/cancer; individual symptoms, cost, etc…)*
   - 1. What or who has the most influence on motivating patients to quit?
     2. Are there any cultural beliefs related to reasons why people might smoke or their efforts to quit smoking?

**III. Putting it all together: LDCT Screening and Tobacco Cessation Shared Decision Making**

1. Thinking about your current tobacco cessation counseling efforts, I would like to hear about what influence you think LDCT screening might have on eligible patients (55-74; 30 pack years, etc…)?
   - 1. How would you integrate LDCT into your efforts to help patients to stop smoking?
     2. Would you anticipate different levels of receptivity from either current smokers or those that quit but are still eligible for LDCT?
     3. Should the provider be the one discussing LDCT with patients? What about other staff? When should it be brought up?

1. An important goal of our project is to help both patients and providers by developing aids to assist with decision making for LDCT screening. As we’ve discussed, LDCT screening is recommended for patients who meet certain criteria but ultimately it’s up to them. Some people refer to these as “preference sensitive decisions.” What is the best way to help patients make decisions in these situations?
2. Thinking more about decision aids, what information or resources would be most helpful to you and your patients?
   1. In what format—written brochures/pamphlets, web-based, other?
   2. When should these decision aids be distributed and discussed with patients?
